# Medical Women at the Johns Hopkins University

**Published:** 1891-02-07

**Authors:** 


### The Hospital

A Weekly Journal of

Science, Medicine, Nursing, and Philanthropy.

*For Hospitals, Asylums, Infirmaries, Dispensaries, Medical Practitioners, Students, Nurses, and the Charitable Public.*

Vol. IX.—No. 228.] [February 7, 1891.

### Medical Women at the Johns Hopkins University.

The women students of medicine in America are to have what they will consider their full rights at last. The trustees of the Johns Hopkins University have decided that the medical school of that institution, which will shortly be ready for its teaching work, shall be open to women "on the same terms as men." In order to secure this right, certain women in America have offered to the trustees, and the offer has been accepted, the sum of 100,000 dollars, by way of an additional endowment fund. Money has thus succeeded where argument has so often failed. It would have been more to the honour of human nature if the success had been due to argument rather than to money.

The step thus taken ought to be considered both separately and as part of a general movement. In itself it will, not unnaturally, be regarded as a triumph by those women who have long toiled and fought for such a consummation. Whilst the battle was in full career there was little use in appealing to the combatants to consider the merits of their cause. But now that victory sits on their helm it is incumbent upon them to value the spoils. What is it that the women of America have gained? And will it be an advantage or an injury to them?

It may be granted at once that there will be some advantages for women in pursuing their medical studies in exactly the same way and on precisely the same terms as men. They will, to a certain extent, learn to see things with men's eyes and to reason about them with men's minds. Moreover, the teachers who teach men anatomy and physiology do not speak with "bated breath," as they do when they are addressing women. Those women medical students who sit in the lecture-rooms with men, and dissect the same bodies with them in the dissecting-rooms, will have all the facts of anatomy and physiology, and all the varieties and incidents of diseases, laid bare before them without any of the checks and veils which an old-fashioned conventionalism might and would suggest. By anatomical, physiological, and pathological science at the Johns Hopkins University sex will be not merely ignored, but absolutely unknown. All this is quite plain and evident, and it is well that no bones whatever should be made about it; but that all who have anything to do with the subject should see the facts, bald and naked, as they actually are. In one part of America, and at an institution which its original founder designed to be a model University for all countries, one of the first steps taken is to utterly abolish sex; and this step has been taken, not as might have been supposed, at the bidding or instigation of men, but at the instigation and bidding of women.

The advantages of the new departure we have admitted. Are there any disadvantages? And, if so, what are they? St. Paul sometimes deprecated the too eager and adverse criticism of those to whom he wrote by pleading the infirmities of his humanity: "I speak as a man," said he. And the present writer is bound to admit that he has the misfortune to belong to the inferior—that is, to the masculine order of sex. That he cannot help. If he gave vent merely to his feelings he would say that he hated and abominated the idea of women sitting side by side with men in the anatomy and physiology class rooms, and working in the same dissecting-room, perhaps at the very same table, in the dissection of human bodies of both sexes. But who gives way to feeling and impulse when addressing women, in these days of scientific advancement? Reason, severe and stern; and logic, perfect and unassailable as chain armour; these are the only weapons and accoutrements with which woman can now be approached by man. Talk not to her of sex! If in the beginning "God created them male and female," it is clear that He never contemplated the scientific American woman of the present period. If He had He would have created them neither male nor female, but flint figures, hollowed out as to the head, and with eyes coldly illuminated by the electric light.

But now, a little uncontrollable feeling having been indulged, reason must be returned to, and the new departure calmly and dispassionately considered. Is it well for women, in the matter of medical education, to be as men are? It is not a question of their being the equals of men: that we do not deny their claim to be. But is it well that they should insist upon being absolutely and identically the same? It certainly is not. Moreover, they cannot be the same. A woman can never be anything but a woman. But even if she could, it is quite certain that a man can never be anything but a man. That is perhaps an aspect of the question which the American women who have raised their hundred thousand dollars, and the University trustees who have accepted the money, have overlooked. *Honi soit qui mal y pense* by all means; and we are quite aware also that to the pure all things are pure, and that to the high and noble man, as to the high and noble woman, St. Paul's argument about the superior honourableness of uncomely parts is absolutely convincing. But what sort of human nature is it we have to deal with at Universities and medical schools? Is it altogether models of chivalrous purity who frequent such seats of learning? It is neither more nor less than the average young man at the age of nineteen to twenty-four. And what is the average young man at that age? A monster? No; but certainly a man! And if all history, sacred and profane, and all experience show anything at all, they show that at that period of life the prayer most needed to be uttered by young men is—"Lead us not into temptation."

The Johns Hopkins trustees must consider what it is they are about to do. They are not agreeing to open their medical lectures and dissecting rooms to a dozen or a score of women of plain appearance, severe character, and mature age. If they were, the question would not be one which required more than five minutes' discussion. But what is the fact? They are preparing to make it part of the constitution of their University that the medical and scientific lectures and the dissecting rooms shall be open to women on exactly the same terms as men. And to how many women, and to what sort of women? To all the women in America, if they choose to come, whatever be their age, and whatever their suitableness or unsuitableness for being placed in such a position of temptation and danger. We said, at the outset, that the question demanded to be considered as part of a general movement. We do not hesitate now to say that the new departure is a menace, not only to the family life, but also to the morals of the whole educated population of America. No doubt the trustees of the Johns Hopkins University are aware of what happened in Switzerland, and of the action taken by the Russian Government, in consequence, to prohibit altogether any attempt on the part of Russian young women to study medicine side by side with men. This one fact is worth reams of argument. If the Johns Hopkins trustees do not wish to bring a noble undertaking into ignominy, they had better reconsider their position while there is yet time.

Our sympathies, as readers of The Hospital know well, are entirely with women in their efforts to emancipate themselves from a disabling conventionalism, and to claim their rights as intellectual beings of independent and original mind. But it is a totally different question when we are asked to sympathise with women in their efforts to make themselves not women. Such efforts are insane, and their permanent success is impossible. Medical women we are willing to have by the score or by the thousand, and we desire for them a training at least equal to that of men, and superior if they can get it; but for the sake of our sisters and our daughters, and for the sake of women generally, and of human nature itself, let us not have this wretched and mad craving after unnaturalnesses and impossibilities. If women are to be doctors, they can only be doctors for women; they can never be doctors for men. In like manner, they should always be taught by women, or, at least, exclusively among women. If it be imperative that they should have men as teachers, let the men be of venerable age and assured character. We are ashamed of the American women that they should have imposed upon us the necessity of writing this article. As for the Johns Hopkins trustees, we cannot help thinking that if they had been a little more chivalrous and a little less hungry for money, they would not have consented to do what they have done either for a hundred thousand or for a hundred million dollars.

